# Marine Yeasts for Biotechnology: Potential Applications and Insights from Comparisons with Terrestrial Yeasts

**DOI:** 10.3390/jof12070522

**Published:** 2026-07-15

**Authors:** Woon-Jong Yu, Ha Young Lee, Hyein Jin, Na Young Choi, Hyeon Gyeong Jeong, Dawoon Chung

**Affiliations:** Department of Biological Application and Technology, National Marine Biodiversity Institute of Korea (MABIK), Seocheon 33662, Republic of Korea; woonjong_yu@mabik.re.kr (W.-J.Y.);

**Keywords:** marine yeasts, terrestrial yeasts, industrial applications

## Abstract

Marine yeasts have attracted increasing interest as potential resources for biotechnology. However, direct comparative studies between marine and terrestrial yeasts remain limited, and the extent to which marine yeasts provide advantages beyond conventional terrestrial yeasts remains unclear. In this review, we synthesize current knowledge of marine yeasts based on approximately 130 publications in the field by organizing the literature into six major themes: comparative studies with terrestrial yeasts, environmental adaptation, metabolic and functional diversity, diversity and distribution, genomic resources, and genetic engineering. Available evidence indicates that marine yeasts possess adaptive traits associated with marine environments, including tolerance to salinity, low temperature, high pressure, and environmental contaminants. These adaptive traits support the use of marine yeasts in seawater-based biofuel production, bioremediation, and the production of enzymes and biosurfactants for saline or other environmentally challenging conditions. We also summarize recent advances in genomics, genetic engineering, and taxonomy that provide useful resources for future studies of marine yeasts. Overall, this review highlights current knowledge of marine yeasts while identifying key gaps that require further comparative investigation with terrestrial yeasts.

## 1. Introduction

Yeasts are widely used in biotechnology because they can be cultivated under defined laboratory conditions, exhibit relatively short growth cycles, and are readily amenable to molecular and genetic manipulation [1]. Consequently, they have been utilized in a broad range of applications, including food fermentation, food and feed production, biocatalysis, recombinant protein production, biocontrol, pharmaceuticals, and fundamental biological research [2].

Since the first isolation of yeasts from the Atlantic Ocean in the 1890s, yeasts have been recovered from diverse marine habitats, including seawater, sediments, marine animals, and marine plants [3]. Like the term “marine fungi”, “marine yeasts” lacks a universally accepted definition [4]. They are sometimes defined as yeasts originating from marine environments that grow better in seawater-based media than in freshwater-based media [5], although many can also grow efficiently under non-marine conditions. Alternatively, marine yeasts could be regarded as yeasts that are repeatedly recovered from marine habitats because they are able to grow and/or sporulate in marine environments, form symbiotic associations with marine organisms, or exhibit genetic or metabolic adaptations to marine environments [6]. This definition has also been adopted in a recent review of marine fungi by Gonçalves et al. (2022) [7].

Marine yeasts have attracted increasing attention as potential resources for biotechnology because of their adaptation to saline, nutrient-limited, and environmentally dynamic marine ecosystems [8]. Compared with conventional terrestrial yeasts, they are often considered to possess greater tolerance to salinity, chemical stress, and environmental fluctuations.

However, it remains unclear whether the adaptive traits of marine yeasts translate into distinct biotechnological advantages over conventional terrestrial yeasts. Marine environments are generally less accessible than terrestrial habitats, and the isolation and cultivation of marine yeasts often require additional effort and cost. Therefore, identifying research areas in which marine yeasts may offer comparable or unique value relative to terrestrial yeasts is important for evaluating their potential as biotechnological resources.

**Figure 1:**
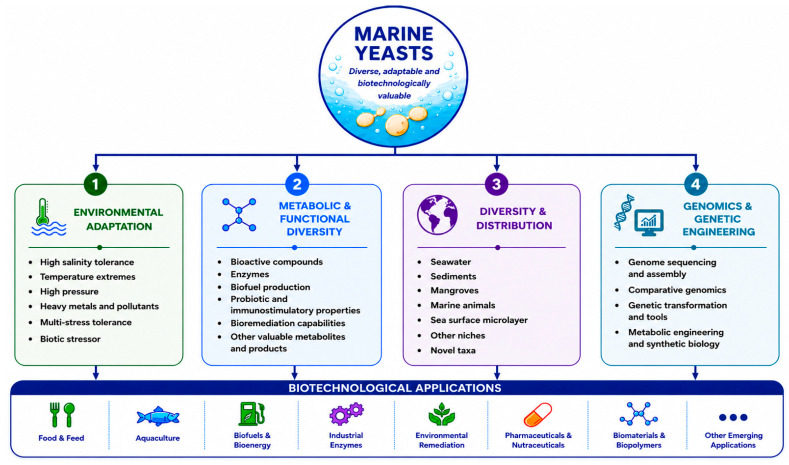
Research on marine yeasts has focused on environmental adaptation, metabolic and functional diversity, diversity and distribution, and genomics and genetic engineering, which together support a wide range of biotechnological applications. Generative AI was used in the preparation of this figure.

To address this question, we reviewed publications on marine yeasts retrieved from PubMed and Google Scholar and organized them into themes relevant to biotechnology. Approximately 130 publications were examined, covering comparative studies with terrestrial yeasts, environmental adaptations, metabolic and functional diversity, diversity and distribution including taxonomic novelty, genomic resources, and genetic engineering. The major research areas discussed in this review are summarized in Figure 1. Together, these thematic perspectives provide a framework for evaluating the research value and potential applications of marine yeasts. Unless otherwise stated, species names used throughout this review follow currently accepted nomenclature based on MycoBank and NCBI Taxonomy.

## 2. Previous Comparative Studies with Terrestrial Yeasts

Comparative studies directly evaluating marine and terrestrial yeasts remain relatively scarce. Two main approaches have been used to assess the applicability of marine yeasts relative to their terrestrial counterparts. The first approach compares marine yeasts with well-characterized reference yeast strains that are widely used in industrial applications, most of which are of terrestrial origin. The compared strains are not necessarily of the same species, as the purpose is to benchmark the characteristics or activities of marine yeasts against established reference strains. These comparisons are performed under comparable laboratory conditions, allowing the characteristics or activities of marine yeasts to be evaluated relative to established terrestrial yeasts. This approach therefore provides an indirect comparison between marine and terrestrial yeasts. The second approach involves direct comparisons between strains of the same species isolated from marine and terrestrial environments, thereby minimizing taxonomic differences and allowing a more equivalent assessment of their physiological and biotechnological traits.

A few studies employing the first approach have been reported. In an early comparative study, the bioethanol-producing capacities of marine-derived *Candida* and *Debaryomyces* strains were compared with those of well characterized terrestrial reference strains (*Saccharomyces cerevisiae* (*S. cerevisiae*) and *Zygosaccharomyces rouxii*), both of which are widely used in industrial fermentation. The comparisons were performed under laboratory conditions. Although ethanol yields of the marine isolates reached only approximately 60–80% of those obtained with the terrestrial reference strains, the marine yeasts exhibited superior salt tolerance, highlighting a potentially valuable process-related trait [9]. A similar comparative framework was later applied to assess γ-aminobutyric acid (GABA) production. In that study, a marine-derived *Pichia* strain was compared with *Saccharomyces* and *Candida* strains used in the food industry. The *Pichia* strain produced approximately 18–24-fold more GABA than the *Saccharomyces* strains, while exhibiting GABA productivity modestly higher (approximately 1.7–1.8-fold) than that of *Candida utilis* and *Candida fermentati* strains [10]. Collectively, these limited comparative studies suggest that marine yeasts may exhibit distinct physiological or metabolic traits of potential industrial relevance, although additional comparative studies are needed to determine whether these observations are broadly applicable.

A broader comparison of marine and terrestrial yeasts has also been conducted through studies of extracellular enzymes. Extracellular enzymes produced by marine yeasts, including species of *Aureobasidium*, *Cryptococcus*, *Metschnikowia*, *Pichia*, and *Yarrowia* were compared with homologous enzymes produced by terrestrial yeasts. Industrially important enzymes such as amylases, cellulases, inulinases, lipases, phytases, and proteases were found in some cases to differ in optimal activity conditions, substrate specificity, molecular weight, and gene structure [11]. As the characteristics of individual enzymes and producing strains have already been comprehensively reviewed elsewhere [8,11], only selected examples relevant to the present review are discussed in Section 4.4.

A representative example of the second approach is provided by recent studies on bioethanol production in seawater-based media. Du and colleagues compared the fermentation performance of marine-derived *S. cerevisiae* AZ65 with that of the terrestrial reference strain *S. cerevisiae* NCYC2592. Strain AZ65 exhibited a higher ethanol productivity (1.77 vs. 1.65 g/L/h; *p* = 0.04) in media prepared with reverse osmosis water [12]. Furthermore, when cultivated in seawater-based medium, ethanol production by AZ65 was approximately 50% higher than that of NCYC2592 [12,13]. Considering that the production of one liter of bioethanol may require up to approximately 9800 L of freshwater [14], marine yeasts capable of efficient fermentation under seawater or high-salinity conditions may represent valuable resources for more sustainable and water-efficient bioethanol production.

The same research group also evaluated the tolerance of 166 marine yeasts and 78 terrestrial yeasts to fermentation inhibitors generated during lignocellulosic bioethanol production, including acetic acid, formic acid, furfural, vanillin, and salt. Compared with the inhibitor-tolerant terrestrial strain *S. cerevisiae* NCYC2592, the marine yeasts *S. cerevisiae* AZ65 and *Wickerhamomyces anomalus* (*W. anomalus*) M15 exhibited 48.3% and 78.3% greater salt tolerance, respectively, and 7.1% and 10.5% greater acetic acid tolerance [13]. Relative to the inhibitor-sensitive terrestrial strain *S. cerevisiae* S288C, AZ65 and M15 showed 62.9% and 56.0% higher tolerance to furfural, 128.2% and 174.4% higher tolerance to salt, and 80.7% and 86.4% higher tolerance to acetic acid, respectively. Furthermore, under inhibitory conditions containing furfural, acetic acid, formic acid, and vanillin, the marine yeast *Yamadazyma membranifaciens* (*Y. membranifaciens*) M2 produced approximately 13% more ethanol than NCYC2592. Under seawater fermentation conditions (salt inhibition), the marine yeasts *S. cerevisiae* AZ65, *W. anomalus* M15, and *Y. membranifaciens* M2 produced approximately 1.85–1.96-fold more ethanol than the terrestrial reference *S. cerevisiae* NCYC2592 [13]. Collectively, these limited comparative studies suggest that some marine yeasts can exhibit fermentation performance and stress tolerance comparable to, or in some cases exceeding, those of terrestrial reference strains. Nevertheless, the currently available evidence remains limited, and further comparative studies encompassing a wider taxonomic and ecological diversity of marine and terrestrial yeasts are needed before general conclusion can be drawn.

## 3. Environmental Adaptations and Stress Tolerance

Marine environments encompass a wide range of habitats, some of which are characterized by challenging conditions such as high salinity, elevated pressure, extreme temperatures, intense ultraviolet radiation, or high concentrations of heavy metals [15]. Like other microorganisms, yeasts have evolved diverse adaptive mechanisms to survive under these environmental conditions.

Recently, two comprehensive reviews containing extensive information on yeasts that survive in extreme environments were published [16,17]. They described current knowledge on yeasts tolerant to salinity, osmotic stress, pressure, temperature extremes, pH stress, radiation, desiccation, heavy metals, and oxidative stress. The extremotolerant and extremophilic yeasts discussed in these reviews originated from diverse environments, including marine, atmospheric, polar, industrial, saline, desert, and rock habitats [16,17]. To specifically evaluate marine yeasts, we conducted an additional literature survey focused on marine-derived taxa, including those not covered in the previous reviews. Topics that substantially overlapped with previous reviews are summarized only briefly with appropriate citations.

### 3.1. High Salinity

Salinity is one of the most defining environmental factors in marine ecosystems and a major selective pressure shaping microbial life. The average salinity of seawater is approximately 35 PSU (approximately equivalent to 35 g of dissolved salts per kg of seawater), although substantial spatial and temporal variations occur depending on evaporation, freshwater input, depth, and geographic location [18].

Marine yeasts have evolved multiple strategies to withstand salinity and osmotic stress, including ion homeostasis, accumulation of compatible solutes (e.g., glycerol), and membrane remodeling [19,20]. A representative example is *Debaryomyces hansenii* (*D. hansenii*), a euryhaline yeast widely used as a model for salinity and osmolarity adaptation [21]. This species has frequently been isolated from marine environments [22,23]. To adapt to changes in salinity, marine *D. hansenii* strains adjust plasma membrane lipid composition, including altered sterol-to-phospholipid ratios and fatty acid unsaturation [22].

Along with *D. hansenii*, black yeasts are another marine yeast group widely used in studies of halotolerance. Black yeasts include *Hortaea werneckii* (*H. werneckii*), *Neophaeotheca triangularis*, and *Aureobasidium pullulans*, which have been isolated from various marine environments [19,24]. The most extensively studied species, *H. werneckii* is able to grow across an exceptionally wide salinity range, from media without added NaCl to saturated conditions (32% NaCl, *w*/*v*), with optimal growth observed at approximately 6–17% NaCl [25]. How black yeasts adapt to high-salinity environments has been well summarized in two review articles previously published [19,20]. Briefly, black yeasts adapt to hypersaline environments through four major mechanisms: accumulation of compatible solutes in the cytosol to maintain osmotic balance, cell wall melanization that enhances intracellular glycerol retention, maintenance of ion homeostasis through diverse metal cation transporters, and adjustment of plasma membrane composition and fluidity in response to salt stress [19,20].

The ability to tolerate elevated salinity is not only ecologically important but also attractive for biotechnology. Halotolerant yeasts can grow and remain metabolically active under conditions that inhibit many conventional microorganisms, thereby reducing contamination risks and enabling processes based on seawater or saline feedstocks. Consequently, they have attracted attention for applications in biofuel production, food and feed processing, saline biorefineries, and biocatalysis [26].

One example is the use of marine yeasts in seawater-based bioethanol production, as described above [12,13]. Another example is the marine-derived halotolerant *D. hansenii* strain Mo40, which exhibited high growth rates and biomass yields in seawater-based media supplemented with low-cost nutrients [27]. The resulting biomass contained high levels of protein and lipids and exhibited phytase activity, suggesting potential value as a food or feed ingredient.

Overall, halotolerance has been one of the most extensively studied biological characteristics of marine yeasts and remains one of their most promising traits for biotechnological applications. Nevertheless, further comparative studies are needed to determine whether the extent and mechanisms of salt adaptation consistently distinguish marine yeasts from their terrestrial counterparts.

### 3.2. Temperature Extremes

The tolerance of marine yeasts to temperature extremes has been associated predominantly with low temperatures rather than high temperatures. Although yeasts have been isolated from deep-sea hydrothermal vent ecosystems, which may contain hydrothermal fluids reaching 150–400 °C, their adaptation to elevated temperatures has rarely been investigated [28].

Psychrophilic and psychrotolerant organisms have evolved a range of adaptive mechanisms that enable them to survive and remain active in low-temperature environments, typically spanning from −20 to 20 °C [29]. Most psychrophilic or psychrotolerant marine yeasts reported to date have been isolated from Antarctic environments (Table 1) [30,31,32,33,34,35,36,37,38,39,40,41]. Research on these yeasts has focused largely on cold-active enzymes, including amylases, α-glucosidases, β-galactosidases, lipases, phytases, proteases, and xylanases [42,43]. These enzymes have attracted increasing interest because they enable energy-efficient processing and help preserve heat-sensitive compounds during industrial processes. For example, the Antarctic marine yeast *Glaciozyma antarctica* (*G. antarctica*) produces several cold-active enzymes, including esterase and chitinase. Its esterase shows maximal activity at 10 °C and retains more than 50% of its activity between 0 and 30 °C, whereas its chitinase exhibits optimal activity at 15 °C and remains active over 5–25 °C, reflecting the high catalytic efficiency of these enzymes at low temperatures [29]. Such properties are considered advantageous for industrial processes requiring catalytic activity at low temperatures, including food processing.

In addition to studies on cold-active enzymes, several investigations have examined the physiological mechanisms underlying cold adaptation in marine yeasts. In the psychrophilic yeast *Mrakia frigida* (*M. frigida*) 5A1, growth at lower temperatures was associated with increased membrane lipid unsaturation, particularly higher proportions of linoleic and linolenic acids, which are thought to contribute to maintaining membrane fluidity under cold conditions [44]. In contrast, transcriptomic analysis of the Antarctic marine yeast *G. antarctica* PI12 revealed a broader range of cold-adaptation mechanisms, including the production of antifreeze proteins to prevent ice crystallization, constitutive expression of molecular chaperones and reactive oxygen species (ROS) detoxification genes, and the utilization of nitrite as an alternative terminal electron acceptor under oxygen-limited conditions [45]. These results indicate that cold adaptation in marine yeasts involves not only membrane remodeling but also coordinated regulation of multiple molecular pathways, including antifreeze protein production, oxidative stress responses, and energy metabolism.

Taken together, these findings suggest that marine yeasts employ multiple complementary strategies to survive at low temperatures. Membrane remodeling, oxidative stress responses, and molecular chaperones appear to represent common cold-adaptation mechanisms among yeasts [46], whereas the contribution of antifreeze proteins has so far been demonstrated only in *Glaciozyma* yeasts isolated from Antarctic environments [47]. Further comparative studies are needed to determine how widely these mechanisms are conserved among marine yeasts.

### 3.3. High Pressure

Because a large proportion of the ocean is exposed to hydrostatic pressures around 38 MPa or higher, compared with approximately 0.101 MPa at sea level, marine environments represent one of the major habitats in which piezophilic and piezotolerant microorganisms can be found [48,49]. These microorganisms are capable of sustaining growth and metabolic activity under elevated hydrostatic pressure and have primarily been isolated from deep-sea habitats, including marine sediments and deep waters [16]. Consequently, they have attracted attention as potential sources of pressure-tolerant biocatalysts and biomolecules for high-pressure industrial processes, including food pasteurization and food processing.

Despite their potential, only a few studies have investigated hydrostatic pressure tolerance in marine yeasts, and research on this topic remains limited. Since then, studies on pressure adaptation have focused primarily on filamentous fungi rather than marine yeasts [50], leaving the mechanisms of hydrostatic pressure adaptation in marine yeasts largely unexplored.

One of the earliest studies on hydrostatic pressure tolerance in marine yeasts demonstrated that *D. hansenii*, *R. mucilaginosa*, and *Rhodotorula sphaerocarpa* (*R. sphaerocarpa*) were capable of growth under simulated deep-sea conditions. All three species grew at pressures up to 20 MPa, and *R. mucilaginosa* and *R. sphaerocarpa* remained capable of growth at 40 MPa [51]. More recently, hydrostatic pressure tolerance was systematically evaluated in 12 yeasts isolated from deep-sea hydrothermal vents by measuring biomass (OD_600_ and ergosterol content), ribosomal activity, and morphological changes under atmospheric pressure, 24.5 MPa, and 60 MPa. Based on these physiological responses, most ascomycetous yeasts studied (*Candida* spp. *D. hansenii*, *H*. *werneckii*, *Meyerozyma guilliermondii* (*M. guilliermondii*)) were classified as piezosensitive, whereas the basidiomycetous yeasts *R. mucilaginosa* and *Rhodotorula diobovata* were classified as piezotolerant because they maintained growth and metabolic activity even at 60 MPa [52].

Overall, the limited studies available indicate that hydrostatic pressure tolerance differs among marine yeast species, although it remains unclear whether these differences reflect general phylogenetic patterns or species-specific adaptations. Further investigations integrating comparative genomics, transcriptomics, and physiological analyses will be required to elucidate the molecular mechanisms underlying hydrostatic pressure adaptation and to assess the biotechnological potential of piezotolerant marine yeasts.

### 3.4. Environmental Pollutants and Multi-Stress Tolerance

Marine ecosystems are continually exposed to a variety of contaminants, including heavy metals, metalloids, and other toxic compounds derived from industrial discharge, agricultural activities, urbanization, and natural geological processes [53]. These pollutants can accumulate in coastal and estuarine environments and may disrupt surrounding ecosystems over time [54]. Microorganisms capable of tolerating and transforming such contaminants are therefore considered important resources for bioremediation. Among them, marine yeasts have attracted increasing attention because of their ability to survive and function under contaminated marine conditions [55].

Several marine yeasts have been reported to tolerate and transform heavy metals. *Debaryomyces hansenii* J6 isolated from a Swedish estuary is tolerant to heavy metals and increases riboflavin production in response to cobalt (II) [56]. Similarly, two *Yarrowia* strains Idd1 and Idd2 isolated from mercury (Hg)-polluted estuarine water showed mercury resistance to 32 mg/L Hg [57,58]. These strains were able to remove more than 97% of Hg from the medium through bioaccumulation, volatilization, and micro-precipitation. In addition, *Yarrowia lipolytica* (*Y. lipolytica*) strains NCIM 3589 and *Yarrowia bubula* NCIM 3590, isolated from oil-polluted seawater and coastal water, respectively, exhibited tolerance to various heavy metals and metalloid, including arsenic, chromium, copper, lead, nickel, and zinc ions [59].

Metalloids can also represent important environmental contaminants in marine ecosystems. Selenium and tellurium oxyanions, particularly selenite and tellurite, are highly toxic forms frequently associated with industrial wastewater and mining effluents [60]. *Rhodotorula mucilaginosa* strains Rm-1A, Rm-13B, and Rm-30B, isolated from salt-marsh sediments, exhibited high resistance to both selenite and tellurite [61]. In addition to resistance, these strains converted nearly 95% of 0.7 mM tellurite into particulate tellurium nanoparticles while also transforming substantial amounts of selenite into amorphous elemental selenium nanoparticles [62].

Beyond metals and metalloids, marine yeasts have also demonstrated tolerance to organic pollutants. The seawater-derived *D. hansenii* strain Y7426 remained viable at benzo(a)pyrene (BaP) concentrations of up to 100 ppm and degraded nearly 70% of the compound within six days [63].

In addition to tolerance toward individual pollutants, several marine yeasts exhibit resistance to multiple environmental stresses. The mangrove sediment-derived yeast *M. guilliermondii* GXDK6 tolerated high salinity (up to 12% NaCl, 18% KCl, and 18% MgCl_2_), manganese concentrations of approximately 5500 ppm, and copper concentrations of up to 1400 ppm, while removing approximately 27% of dissolved copper after 96 h [64,65]. To better understand the molecular basis of this remarkable stress tolerance, multi-omics analyses were subsequently performed. Salt tolerance was associated with the regulation of osmotic stress signaling (e.g., *YPD1*), antioxidant defense (*CTT1* and *SOD*), protein synthesis, and amino acid and carbohydrate metabolism, together with the accumulation of osmoprotective metabolites such as β-alanine and D-mannose [65]. Copper tolerance was associated with the coordinated regulation of copper transport (*CCC2* and *CTR3*), glutathione metabolism, antioxidant defense, and carbon metabolism, providing insight into the molecular mechanisms underlying adaptation to heavy metal stress [64].

Another mangrove-derived yeast, *Pichia kudriavzevii* (*P. kudriavzevii*) HJ2, exhibited tolerance to acidic (pH 2.0) and bile salt (3%) conditions while producing volatile aroma compounds, including isoamyl acetate [66]. More recently, this strain was also shown to efficiently remove ammonia nitrogen (NH_3_/NH_4_^+^) under high-ammonia conditions, suggesting potential applications in aquaculture systems, industrial wastewater treatment, and eutrophic coastal environments [67].

### 3.5. Biotic Stressors

In addition to adaptation to abiotic stresses, interactions with other marine microorganisms may also contribute to the evolution of marine yeasts. A recent study demonstrated that prolonged interaction with the free-living amoeba induced multiple phenotypic variants in the marine- and brackish-associated *Candida haemulonii* complex, including enhanced biofilm formation, filamentation, extracellular enzyme production, and increased virulence-associated gene expression [68]. These findings suggest that interactions with environmental predators may act as selective forces driving phenotypic diversification in marine yeasts.

Similar interactions have been proposed to influence the evolution of virulence in terrestrial yeasts, including *Cryptococcus neoformans* [69] and *Candida albicans* [70], in which resistance to amoebal predation is thought to have contributed to traits that also enhance survival in animal hosts. Therefore, microbial interactions may represent an important but currently underexplored aspect of marine yeast adaptation, linking ecological adaptation with the emergence of virulence-related phenotypes.

Taken together, these studies demonstrate that marine yeasts have evolved diverse adaptive strategies to cope with both abiotic and biotic challenges, supporting their potential for biotechnological applications under saline and environmentally stressful conditions. Nevertheless, comparative studies between marine and terrestrial yeasts remain limited. Although both groups have demonstrated tolerance to a variety of environmental stresses and contaminants such as heavy metals [71,72,73], it remains unclear whether marine yeasts possess distinct or superior adaptive mechanisms or primarily share similar stress response strategies while functioning under saline marine conditions. Further comparative physiological and genomic studies will be required to clarify the unique adaptive features of marine yeasts and their potential advantages for biotechnology.

## 4. Metabolic and Functional Diversity

Marine yeasts have been investigated as sources of diverse metabolites, enzymes, and bioactive products with potential industrial applications. Several review articles have summarized the potential biotechnological applications of marine yeasts, including the production of extracellular enzymes, killer toxins, siderophores, polysaccharides, single-cell proteins and oils, bioethanol, and nanoparticles [5,8,11,74,75]. However, most of these reviews were published nearly a decade ago and therefore do not reflect the substantial increase in marine yeast research reported in recent years.

To complement these earlier reviews, this section focuses on studies published since 2016 and highlights recent advances in the metabolic and functional diversity of marine yeasts, including probiotic and bioactive properties, bioenergy-related metabolites, bioremediation, enzymes, and other industrially valuable metabolites and products.

### 4.1. Probiotic, Immunostimulatory, and Bioactive Properties

Recent studies have increasingly highlighted the potential of marine yeasts as probiotics and immunostimulants for aquaculture species. Marine yeast strains, including *Y. lipolytica* and *D. hansenii* isolated from seawater and solar saltern environments, have been reported to enhance innate immune responses, antioxidant activities, intestinal health, and resistance against bacterial pathogens such as *Vibrio parahaemolyticus* and *Escherichia coli* in fish and shrimp [76,77,78,79]. The immunostimulatory activity of *Y. lipolytica* has been attributed primarily to β-glucans in the yeast cell wall, which act as microbe-associated molecular patterns (MAMPs) to activate host immune signaling pathways [76,78,79]. The beneficial effects of *D. hansenii* are mainly associated with the activation of antioxidant defense pathways, particularly through increased superoxide dismutase activity, which protects immune cells from oxidative stress and enhances phagocytic and antimicrobial responses [77].

In addition, β-glucans derived from marine yeasts, including *D. hansenii* and *Sterigmatomyces halophilus* (*S. halophilus*), were shown to stimulate immune-related signaling pathways in goat and Pacific red snapper peripheral blood leukocytes, respectively, while also enhancing leukocyte activity, further supporting their potential use as functional feed additives [80,81,82]. The immunostimulatory effects of *D. hansenii* β-glucans are mediated through activation of Dectin-1- and Toll-like receptor-associated innate immune signaling, resulting in enhanced cytokine production and leukocyte function [80,81]. β-Glucans from *S. halophilus* enhanced the expression of pro- and anti-inflammatory cytokine genes, indicating activation of host immune signaling, although the upstream signaling pathways responsible for these responses have not yet been elucidated [82].

Beyond their effects on host health, some marine yeasts have demonstrated beneficial functions in aquaculture environments. For example, *R*. *sphaerocarpa* isolated from aquaculture seawater promoted ammonia nitrogen removal and suppressed the growth of bacterial pathogens when introduced into shrimp culture systems [83], suggesting potential applications in both aquaculture and aquaculture wastewater treatment. The observed ammonia removal was attributed to ammonium assimilation into yeast biomass rather than nitrification, as no nitrite accumulation was detected [83].

The beneficial effects of marine yeasts have also been reported in livestock. Dietary supplementation with marine red yeast (species not specified) improved small intestinal homeostasis and laying performance in chickens. At the molecular level, marine red yeast promoted intestinal barrier function through the upregulation of tight junction proteins, enhanced autophagy, reduced epithelial apoptosis and inflammatory responses, and reshaped the gut microbiota toward increased short-chain fatty acid production, thereby restoring intestinal homeostasis [84]. Similarly, supplementation with *Rhodotorula paludigena* reduced the incidence of diarrhea and enhanced immune responses in early-weaned lambs. These effects were associated with enhanced mucosal immune function, suppression of intestinal inflammatory responses, and modulation of the gut microbiota [85].

Overall, current evidence indicates that marine yeasts possess diverse probiotic and immunomodulatory properties that may benefit both aquaculture species and livestock. However, most studies have focused on a limited number of yeast species and experimental models, and further comparative studies are needed to determine whether these beneficial effects are broadly applicable and reproducible under commercial production conditions.

### 4.2. Biofuel Production

Marine yeasts have attracted attention as potential producers of biofuels because they can utilize seawater and marine biomass-derived feedstocks, thereby reducing freshwater demand in biorefinery processes.

Bioethanol production is the most extensively studied biofuel application of marine yeasts. As described in Section 2, *S. cerevisiae* AZ65 produced up to 93.5 g/L ethanol with a productivity of 2.49 g/L/h in seawater-based medium and outperformed a terrestrial reference strain under the same conditions [12,13]. Similarly, *W. anomalus* M15 produced up to 73 g/L ethanol from concentrated green algal hydrolysates, while *Wickerhamomyces subpelliculosus* ZE75 exhibited the highest ethanol production per unit biomass when cultivated in seawater-based medium [86,87,88]. These studies demonstrate the suitability of marine yeasts for ethanol production from seawater-based and marine biomass-derived substrates.

Marine yeasts have also been investigated as oleaginous microorganisms for biodiesel production. *Rhodosporidium* sp. TJUWZ4 accumulated lipids up to 44% of biomass under optimized conditions [89]. Likewise, *Rhodosporidiobolus fluvialis* Y2 accumulated lipids enriched in polyunsaturated fatty acids when grown on mannitol, a major carbohydrate component of brown algae, suggesting the potential use of marine biomass for biodiesel production [90].

### 4.3. Bioremediation

Marine environments are exposed to diverse anthropogenic pollutants, including hydrocarbons, halogenated organic compounds, excess nitrogen, plastics, radionuclides, and metalloid oxyanions [91]. Recent studies suggest that marine yeasts may serve as useful bioremediation agents because of their ability to tolerate, transform, or remove a wide range of contaminants [92,93,94,95,96,97,98,99].

Among the reported species, *Y. lipolytica* NCIM 3589 is one of the most extensively studied marine yeasts for xenobiotic degradation. Isolated from oil-contaminated seawater, this strain degraded brominated aromatic compounds such as bromobenzene and possessed a bifunctional epoxide hydrolase/dehalogenase involved in the transformation of structurally diverse bromoorganic compounds [92,93].

Marine yeasts have also shown potential for the remediation of petroleum hydrocarbons. The halotolerant yeast *Candida tropicalis* B degraded crude oil and various aliphatic and aromatic hydrocarbons [94]. In addition, *Y. lipolytica* LMS 24B produced a thermostable biosurfactant with a high emulsification index, indicating potential utility in oil-contaminated marine environments [95].

Nitrogen removal represents another potential application. *R. sphaerocarpa* YLY01 removed approximately 86% of ammonia nitrogen while suppressing the growth of *Vibrio* pathogens [83]. Likewise, *W. anomalus* RZWP01 efficiently removed nitrite and ammonium, whereas *P. kudriavzevii* HJ2 completely removed 300 mg/L ammonia nitrogen within one day under optimal conditions [66,96]. These findings highlight the potential use of marine yeasts in aquaculture and wastewater treatment systems.

Marine yeasts have also been investigated for the remediation of radionuclides and emerging contaminants. *Y. lipolytica* NCIM 3589 tolerated uranium through biosorption and biomineralization mechanisms [97,98]. In addition, *R. mucilaginosa* 11602 degraded polyethylene and assimilated polyethylene-derived carbon into biomass [99], while other *R. mucilaginosa* strains have been reported to transform toxic selenium and tellurium oxyanions into less bioavailable forms [61,62].

### 4.4. Enzymes

Fungi, including yeasts, constitute an important source of industrially relevant enzymes with applications in food processing, biocatalysis, pharmaceutical manufacturing, and detergent industries [100]. Like terrestrial yeasts, marine yeasts inhabit environments that experience fluctuations in temperature, oxygen content, and nutrient availability [101]. However, marine environments impose the additional challenge of persistent salinity, which may contribute to the development of enzymes with salt tolerance and activity under diverse reaction conditions [102]. Although evidence remains limited to a relatively small number of enzymes and species, these characteristics suggest considerable potential for industrial applications. Marine yeast-derived enzymes and their producing strains have been reviewed previously [8,11]. Here, we focus on representative studies published since those reviews.

Marine yeasts have been identified as producers of proteases, one of the most commercially important classes of industrial enzymes. Proteases have broad applications in the detergent, food, leather, waste management, and pharmaceutical industries [103]. The Antarctic marine yeast *R. mucilaginosa* L7 was reported to produce extracellular proteases, and optimization of cultivation conditions significantly enhanced enzyme production [104]. More recently, an alkaline protease from *Lodderomyces orthopsilosis* AKB-1 isolated from mangrove sediment exhibited antimicrobial activity against pathogenic bacteria, including *Bacillus cereus*, and demonstrated stain-removal performance comparable to that of commercial detergents [105]. These characteristics suggest potential applications in detergent formulations and other industrial bioprocesses.

Laccases are another enzyme group of interest in marine yeasts because they catalyze the oxidation of a wide range of phenolic and aromatic compounds, making them valuable for bioremediation, textile dye decolorization, lignocellulose pretreatment, and biomass conversion [106]. A recombinant laccase encoded by the LAC1 gene from the marine-derived yeast *Aureobasidium melanogenum* (*A. melanogenum*) 11-1 effectively decolorized synthetic dyes [107].

Marine yeasts have additionally been identified as producers of carbohydrate-degrading enzymes. Among them, amylases are of particular industrial interest because they are widely used for starch hydrolysis in food, brewing, bioethanol, and other carbohydrate-processing industries [108]. The marine alga-derived yeast *Sporobolomyces japonicus* PH-Gra1 exhibited significant amylolytic activity, and the enzyme remained active under low to moderate salinity conditions (0–3% NaCl) [109]. Such salt-tolerant amylases may be advantageous for starch-processing and other carbohydrate-based industrial processes conducted under saline conditions.

### 4.5. Other Industrially Valuable Metabolites and Products

Marine yeasts produce a wide variety of value-added metabolites and products with potential applications in the food, pharmaceutical, cosmetic, and materials industries. These include carotenoids, biosurfactants, specialty chemicals, biodegradable polymers, and nutritionally valuable lipids, further demonstrating the metabolic versatility of marine yeasts.

Carotenoids are commercially important natural pigments because of their antioxidant activity and widespread applications in the food, feed, cosmetic, and nutraceutical industries [110]. Marine yeasts have been reported as producers of these compounds. For example, *R. mucilaginosa* cultivated on media prepared from extracts of the brown macroalga *Macrocystis pyrifera* produced commercially important carotenoids, including lycopene, β-carotene, and astaxanthin [111]. Similarly, *Rhodotorula* sp. KSB1 isolated from mangrove environments produced carotenoid pigments exhibiting both antioxidant activity and antibacterial activity against *Escherichia coli*, *Listeria monocytogenes*, and *Staphylococcus aureus* [112].

Biosurfactants are biodegradable surface-acting compounds with applications in bioremediation, cosmetics, pharmaceuticals, food processing, and agriculture [113]. Marine yeasts have also been investigated as producers of biosurfactants and glycolipid-based compounds. *Rhodotorula mucilaginosa* cultivated on seaweed extracts produced sophorolipids with antibacterial activity against both *E. coli* and *S. aureus* [114]. In addition, the mangrove-derived yeast *Moesziomyces bullatus* XM01 efficiently produced mannosylerythritol lipids, a class of glycolipid biosurfactants with potential applications in cosmetics, pharmaceuticals, and nanotechnology [115].

Marine yeasts also produce bioactive proteins with potential industrial applications. Yeast killer toxins are proteins or glycoproteins that exhibit antagonistic activity against susceptible microorganisms and have attracted interest for applications in the food and feed industries as well as in the preharvest and postharvest biocontrol of plant pathogens [116]. The psychrotolerant marine yeast *M. frigida* 2E00797 produces a killer toxin active against *M. bicuspidata*, with maximal activity at 15 °C [36]. In addition, the marine-derived yeast *Pichia anomala* YF07b, isolated from the gut of a sea squirt, produces a β-1,3-D-glucanase killer toxin with maximal activity at 40 °C [117]. These studies demonstrate the diversity of killer toxins produced by marine yeasts and their potential for biocontrol and other industrial applications.

Several marine yeasts have additionally been explored for the production of industrially relevant chemicals such as glycerol. Glycerol is an important platform chemical widely used in the food, cosmetic, pharmaceutical, and chemical industries [118]. UV-mutagenized derivatives of *W. anomalus* HH16 isolated from marine sediment produced up to 73.33 g/L glycerol in seawater-based media, demonstrating the feasibility of glycerol production under saline cultivation conditions [119].

Marine yeasts are also capable of producing polyhydroxyalkanoates (PHAs), a class of biodegradable biopolymers considered sustainable alternatives to petroleum-based plastics [120]. A halophilic *Pichia* sp. TSLS24 isolated from sediment near a livestock wastewater treatment area accumulated PHAs, achieving a PHA content of 43.4% and a concentration of 1.8 g/L under optimized conditions [121]. The resulting polymer exhibited substantial biodegradability in seawater, supporting its potential as an alternative to petroleum-based plastics.

Marine yeasts can serve as a source of nutritionally valuable lipids. Cold-adapted marine yeasts including *R. mucilaginosa* and *Lodderomyces elongisporus* accumulated substantial amounts of polyunsaturated fatty acids, particularly linoleic acid and α-linolenic acid [40]. These fatty acids are of considerable interest because of their nutritional benefits and potential applications in functional foods and nutraceutical products.

Overall, many of the products described above, including bioethanol, carotenoids, glycerol, killer toxins, lipids, and enzymes, can also be produced by terrestrial yeasts [12,116,122,123,124,125,126,127,128]. Therefore, the biotechnological value of marine yeasts does not necessarily lie in the uniqueness of these products themselves, but rather in their ability to produce them under saline conditions or using seawater and marine biomass-derived feedstocks. These characteristics may reduce freshwater consumption, decrease contamination risks during cultivation, and facilitate bioprocesses under conditions that are challenging for many conventional terrestrial yeasts. However, additional comparative studies are required to determine whether marine yeasts consistently offer advantages over terrestrial counterparts across different industrial applications.

## 5. Diversity and Distribution of Marine Yeasts

**Figure 2:**
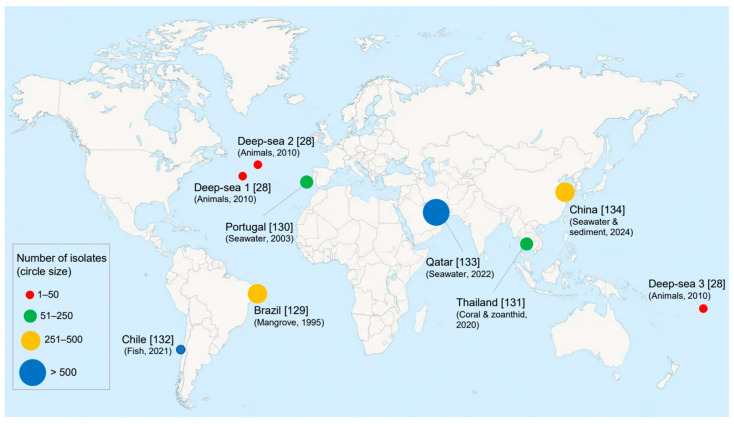
Geographic distribution of representative studies on marine yeast diversity. Sampling locations are shown together with the reported habitat type and year of publication. Circle size represents the approximate number of yeast isolates recovered in each study.

Marine yeasts have been isolated from a wide range of marine habitats, including seawater, sediments, deep-sea environments, mangrove and hydrothermal vent ecosystems, and various marine plants and animals (Figure 2). Several culture-dependent studies revealed that marine environments harbor diverse yeast communities composed of both Ascomycota and Basidiomycota.

One of the earliest surveys of marine yeasts, published in 1995, was conducted in a Brazilian mangrove ecosystem and recovered 252 isolates representing 40 known species and 44 putative novel species [129]. Similarly, seawater samples collected above the Alvares Cabral Trench off Portugal yielded 234 isolates, with basidiomycetous yeasts being more abundant and diverse than ascomycetous yeasts [130].

Subsequent studies expanded the known distribution of marine yeasts to specialized habitats. Culturable yeasts have been isolated from deep-sea hydrothermal vent fauna, including shrimps, mussels, corals, and sponges, with representative genera including *Candida*, *Cryptococcus*, *Debaryomyces*, *Rhodosporidium*, and *Rhodotorula* [28]. Diverse yeast communities have also been reported from corals and zoanthids in the Gulf of Thailand, where four ascomycetous genera (*Candida*, *Meyerozyma*, *Kodamaea*, and *Wickerhamomyces*) and eight basidiomycetous genera (*Vishniacozyma*, *Filobasidium*, *Naganishia*, *Papiliotrema*, *Sterigmatomyces*, *Cystobasidium*, *Rhodotorula*, and *Rhodosporidiobolus*) were isolated. The species with the highest occurrence was *R. mucilaginosa* [131]. In addition, marine fishes harbor diverse gastrointestinal yeasts, including species of *Cystobasidium*, *Diutina*, *Rhodotorula*, *Suzukiozyma*, and *Yamadazyma* [132].

A large-scale survey reported in 2022 further demonstrated the diversity of marine yeasts. In hypersaline coastal waters surrounding Qatar, 842 isolates representing 82 species were recovered, including eleven species newly reported from marine environments [133]. Likewise, a survey published in 2024 recovered 392 isolates from intertidal sediments and seawater in Aoshan Bay, China, representing 20 genera and 43 species. The survey identified 17 species newly reported from marine habitats and revealed marked spatial and temporal variation in community composition [134].

Although dominant taxa vary among habitats, genera such as *Candida*, *Cryptococcus*, *Debaryomyces*, *Rhodosporidium*, and *Rhodotorula* are repeatedly reported across geographically distant marine environments. Most studies of marine yeast diversity have relied on culture-dependent methods, whereas the application of DNA metabarcoding remains limited. Consequently, the currently recognized diversity of marine yeasts likely represents only a fraction of the diversity present in marine ecosystems.

The continuous discovery of novel taxa further supports the view that marine yeast diversity remains incompletely characterized. Over the past three decades, numerous new yeast species and several novel genera have been described from marine environments (Table 2) [135,136,137,138,139,140,141,142,143,144,145,146,147,148,149,150,151,152,153]. Deep-sea ecosystems [136,137,140,141,146,147] have been particularly important sources of novel taxa. However, new species have also been reported from a wide range of other marine habitats, including mangrove-associated habitats [145,150], seawaters [138,142,144,152,153], marine surface microlayers [135], marine sediments [139,148], marine animals (e.g., sponges and corals) [143,149], and marine driftwood [151]. Recent taxonomic studies have further proposed new genera and reclassifications based on multi-locus phylogenetic and genomic analyses [154].

Taken together, these findings indicate that marine habitats remain an important reservoir of yeast diversity. However, current knowledge is still strongly influenced by geographically uneven sampling and the predominance of culture-dependent approaches. Broader geographic surveys combined with culture-independent sequencing and integrative taxonomic analyses will be essential for obtaining a more comprehensive understanding of marine yeast diversity and distribution.

## 6. Genomic Insights into Marine Yeast Adaptation

Advances in genome sequencing technologies have greatly improved our understanding of microbial physiology, evolution, and environmental adaptation. Genomic resources for marine yeasts remain limited compared with those available for terrestrial yeasts. For example, a large-scale whole-genome sequencing study analyzed 1011 terrestrial *S. cerevisiae* isolates collected from diverse sources, including food, humans (clinical samples), soil, freshwater, plants, and animals, to investigate the phenotypic diversity of natural populations [155]. In addition, comparative genomic analyses of twelve food-associated yeast species, including *S. cerevisiae*, have been conducted to elucidate the metabolic pathways involved in volatile flavor production [156], illustrating the extensive application of genomics to terrestrial yeasts.

Draft or complete genome sequences have been reported for several marine yeasts, including *Metschnikowia australis* UFMG-CM-Y6158, *Lizanozyma spartinae* (*L. spartinae*) ARV011, *Metschnikowia zobellii* gsMetZobe1, and *R. sphaerocarpa* GDMCC 60679 [157,158,159,160]. These genomes typically range from approximately 12 to 18 Mb and contain between 4400 and 6100 predicted protein-coding genes. The growing availability of genomic resources is expected to facilitate future studies on the physiology, evolution, and biotechnological potential of marine yeasts.

Comparative genomics has further provided insights into the evolutionary strategies employed by marine yeasts in response to environmental constraints. A representative example is *R. sphaerocarpa* ETNP2018, isolated from the oligotrophic oxygen minimum zone of the eastern tropical North Pacific [161]. Comparative analyses with related marine and terrestrial *Rhodotorula* genomes revealed a streamlined genome characterized by reductions in genes encoding major facilitator superfamily transporters and biosynthetic functions, while core metabolic pathways remained conserved [161]. The observed genome reduction in *R. sphaerocarpa* may represent a strain-specific adaptation to oligotrophic marine environments rather than a general evolutionary strategy of marine yeasts. Additional comparative genomic studies are required to determine whether genome streamlining is widespread among marine yeasts.

Overall, the currently available genome sequences demonstrate the potential of genomics to elucidate the molecular basis of marine adaptation and to identify genes relevant to future biotechnological applications. However, genomic resources for marine yeasts remain limited in both taxonomic and ecological coverage. Expanding genome sequencing and comparative genomic analyses across diverse marine yeast taxa will be essential for gaining a broader understanding of their evolution, environmental adaptation, and biotechnological potential.

## 7. Genetic Transformation and Metabolic Engineering of Marine Yeasts

The development of genetic transformation systems is an important prerequisite for functional genomic studies and metabolic engineering. Although molecular tools for marine yeasts remain less developed than those available for conventional industrial yeasts, several studies have demonstrated that marine yeasts can be genetically manipulated and utilized as potential microbial cell factories.

Early studies on the halotolerant marine yeast *D. hansenii* established transformation systems and expression vectors, providing foundational tools for gene function analysis and heterologous gene expression [162]. More recently, a transformation system was successfully developed for the marine yeast *L. spartinae* YMxiao, further expanding the range of genetically tractable marine yeast species [159].

Marine-derived yeasts have also been explored as hosts for recombinant protein production. For example, *Y. lipolytica* SWJ-1b isolated from the gut of a marine fish was successfully engineered for heterologous protein expression and surface display. In addition, its high crude protein content suggests potential utility as a single-cell protein source while serving as a platform for recombinant protein production [163].

Metabolic engineering has likewise been applied to enhance the production of industrially relevant metabolites. In the mangrove-derived yeast *A. melanogenum* P16, heterologous expression of the *INU1* gene encoding an inulinase from *Kluyveromyces marxianus* significantly increased extracellular inulinase activity and enabled the efficient conversion of inulin into pullulan [164]. The engineered strain produced substantially higher levels of pullulan from inulin than the parental strain, demonstrating the feasibility of improving biopolymer production through genetic modification of marine-derived yeasts.

Given that numerous genetically engineered terrestrial yeasts have already been developed for industrial applications, the selection of marine yeasts as targets for genetic engineering should be supported by evidence demonstrating advantages over their terrestrial counterparts. Such evidence may come from comparative studies showing unique or superior biological functions, or from traits that make marine yeasts particularly suitable for saline environments, seawater-based bioprocesses, or the utilization of marine biomass-derived feedstocks. Identifying marine yeasts with these distinctive characteristics will provide a stronger rationale for future metabolic engineering and the development of marine yeast cell factories.

## 8. Conclusions

Current evidence suggests that marine yeasts possess adaptive traits associated with life in marine environments, including tolerance to high salinity, low temperatures, hydrostatic pressure, environmental pollutants, and multiple environmental stresses. However, direct comparative studies with terrestrial yeasts remain limited, making it difficult to determine the extent to which these traits are unique to marine yeasts or provide advantages over their terrestrial counterparts. Consequently, these characteristics should not be interpreted as evidence that marine yeasts are universally superior to terrestrial yeasts. Rather, they suggest that marine yeasts may be particularly well suited for industrial processes conducted under saline or otherwise challenging environmental conditions, where conventional terrestrial yeasts may be less suitable.

An important implication of these adaptive traits is their potential use in sustainable bioprocesses that utilize seawater and marine biomass as alternatives to freshwater and terrestrial feedstocks. Marine yeasts capable of efficient growth and production under these conditions could contribute to reducing freshwater consumption, lowering contamination risks, and improving the sustainability of industrial biotechnology.

Future research should focus on identifying marine yeasts that possess demonstrable advantages over terrestrial counterparts for specific applications. Achieving this goal will require additional comparative physiological and genomic studies to clarify the ecological significance of marine adaptation and determine which species or strains are best suited for seawater-based and other sustainable bioprocesses.

## Figures and Tables

**Table 1:** Psychrotolerant or psychrophilic marine yeast.

Marine Yeast Strains	Sources	Cultivation Temperature Tested	References	Notes (Current Name)
Antarctic environments				
*Cystofilobasidium infirmominiatum*	marine sponges	4–23 °C	[30]	
*Glaciozyma antarctica* (*G. antarctica*) PI12	sea ice	4–15 °C	[31]	
*Guehomyces pullulans* 17-1	sediment	10–25 °C	[32]	*Tausonia pullulans* (*T. pullulans*)
*Leucosporidiella creatinivora*	marine sponges	4–23 °C	[30]	*Leucosporidium creatinivorum*
*Leucosporidium antarcticum*	seawater	5–20 °C	[33]	*G. antarctica*
*Leucosporidium scottii* L117	sediment	15 °C	[34]	
*Metschnikowia australis*	marine sponges	4–23 °C	[30]	
*Mrakia blollopsis* SK-4	algal mat of sediment	4–22 °C	[35]	
*Mrakia frigida* 2E00797	sea sediment	10–20 °C	[36]	
*Rhodotorula mucilaginosa* (*R. mucilaginosa*) AN5	sea ice	0–40 °C	[37]	
*R*. *mucilaginosa* JMUY14	sediment	15 °C	[38]	
*R. mucilaginosa* L7	marine alga	15–25 °C	[39]	
*Rhodotorula pinicola*	marine sponges	4–23 °C	[30]	*Cystobasidium pinicola*
Others				
*Lodderomyces elongisporus*	sea fish (*Epinephelus aeneus*)	7–35 °C	[40]	
*Rhodotorula infirmo-miniata*	sea fish	4–20 °C	[41]	*Cryptococcus infirmominiatus*
*R. mucilaginosa*	sea fish (*Epinephelus areolatus*)	7–30 °C	[40]	
*Trichosporon pullulans*	sea fish	4–20 °C	[41]	*T. pullulans*

**Table 2:** Novel yeast taxa isolated from marine habitats and their current taxonomic status.

Yeast Species	Phylum	Habitats/Source	Location	Reference
*Candida neustonensis*	Ascomycota	Sea surface microlayer	Taiwan	[135]
*Candida oceani* (*Yamadazyma oceani*)	Ascomycota	Hydrothermal vent-associated coral, seawater, and fish	Mid-Atlantic Ridge	[136]
*Cryptococcus surugaensis* (*Hannaella surugaensis*)	Basidiomycota	Deep-sea sediment	Japan	[137]
*Cystobasidium halotolerans*	Basidiomycota	Seawater	Qatar	[138]
*Cystofilobasidium josepaulonis*	Basidiomycota	Marine sediment	China	[139]
*Dipodascus tetrasporeus* (*Geotrichum tetrasporum*)	Ascomycota	Deep-sea sediment	Japan Trench	[140]
*Kluyveromyces nonfermentans*	Ascomycota	Deep-sea sediment, clam, and crab	Japan	[141]
*Kondoa qatarensis*	Basidiomycota	Seawater	Qatar	[142]
*Leucosporidium escuderoi*	Basidiomycota	Marine sponge	Antarctica	[143]
*Naganishia qatarensis*	Basidiomycota	seawater	Qatar	[144]
*Nigromyces azzae*	Ascomycota	Mangrove tree	Kuwait	[145]
*Rhodotorula benthica*	Basidiomycota	Deep-sea tubeworm	Pacific Ocean	[146]
*Rhodotorula calyptogenae* (*Cystobasidium calyptogenae*)	Basidiomycota	Deep-sea giant white clam	Pacific Ocean	[146]
*Rhodotorula pacifica*	Basidiomycota	Deep-sea sediment	Pacific Ocean	[147]
*Rhodotorula portillonensis* (*Cystobasidium portillonensis*)	Basidiomycota	Marine sediment	Antarctica	[148]
*Spencerozyma siamensis*	Ascomycota	Soft coral	Thailand	[149]
*Sympodiomycopsis kandeliae*	Basidiomycota	Flowers (Kandelia candel) in mangrove forests	Taiwan	[150]
*Sympodiomycopsis lanaiensis* (*Jaminaea lanaiensis*)	Basidiomycota	Marine driftwood	Hawaii, USA	[151]
*Torulopsis haemulonii* (*Candidozyma haemuli*)	Ascomycota	seawater	Atlantic Ocean	[152]
*Yamadazyma barbieri*	Ascomycota	Hydrothermal vent seawater and coastal water	Mid-Atlantic Ridge	[153]

## Data Availability

No new data were created or analyzed in this study. Data sharing is not applicable to this article. All information analyzed in this review is available in the cited references.
